# Increasing water stress in developing countries

**DOI:** 10.1016/j.xinn.2025.101037

**Published:** 2025-07-12

**Authors:** Yao Li, Gang Zhao

**Affiliations:** 1 School of Geographical Sciences, Southwest University, Chongqing 400715, China; 2 Key Laboratory of Water Cycle and Related Land Surface Processes, Institute of Geographic Sciences and Natural Resources Research, Chinese Academy of Sciences, Beijing 100101, China

## Abstract

Global water scarcity is an escalating issue, with billions of people facing severe water stress and limited access to water for extended periods each year. Climate change and increasing human demand are expected to exacerbate this problem in the coming decades. Reservoirs are critical for managing water resources, yet significant disparities exist between developed and developing countries. In developing regions, although total reservoir storage is increasing, the effectiveness of this storage is diminishing, as reflected in declining normalized storage levels. This indicates that these countries are becoming more vulnerable to water scarcity. In contrast, developed countries show more stable and efficient use of their water storage. The declining per-capita water storage across both developed and developing nations underscores the urgency of addressing water scarcity through improved reservoir management, enhanced conservation efforts, and alternative water sources, especially in regions with rapid population growth and limited resources.

## Main text

Over 2 billion people are experiencing high water stress, and around 4 billion are suffering from severe water scarcity for at least one month each year.^1^ Meanwhile, increasing water demand for industrial and domestic purposes is projected to boost global water use by 20%–33% by 2050 compared to 2010 levels.^2^ Climate change, exacerbated by human activities, is expected to intensify water scarcity both regionally and globally.^3^^,^^4^ Over the past two decades, floods and droughts have caused over 166,000 deaths, affected more than 3 billion people, and incurred nearly $700 billion in economic losses.^5^ In response, reservoirs—designed for functions such as water supply, hydropower, irrigation, and flood control—are becoming increasingly vital in the context of a changing climate, especially in developing countries. Although reservoirs represent only about 4% of the total storage capacity of lakes and reservoirs, they account for 61% of the variability in surface water storage.^6^

**Figure 1:**
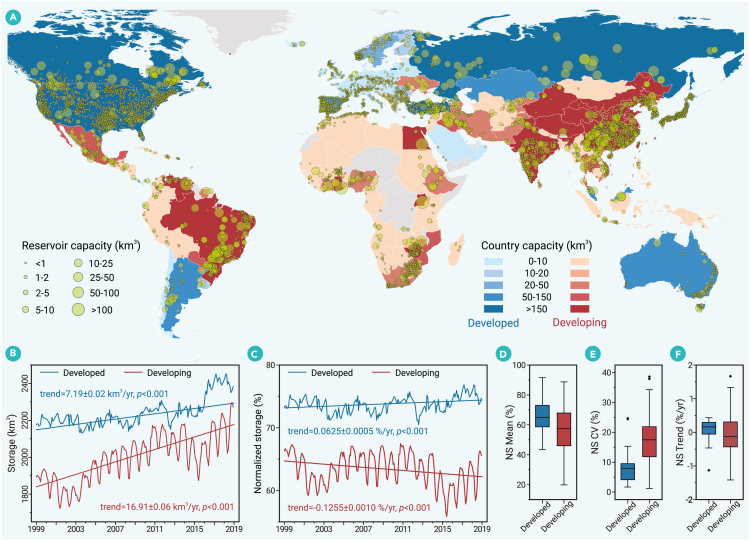
Comparison of reservoir storage variations between developed and developing countries (A) Global reservoir locations and total reservoir capacity by country. (B and C) Trends in (B) total reservoir storage and (C) normalized storage (NS) for developed and developing countries, respectively. (D–F) Comparisons of NS metrics between developed and developing countries, including (D) mean NS values, (E) coefficient of variation (CV), and (F) NS trends from 1999 to 2018. Box ranges represent the upper and lower quartiles, whiskers extend to 1.5 times the interquartile range, and outliers are denoted as dots. Countries are classified as developed or developing based on the United Nations Human Development Index, with a threshold of 0.8 according to the Human Development Report 2020.

Satellite remote sensing provides an unprecedented tool for multi-scale monitoring of inland water quantity, offering key measurements such as surface area, water level, and storage variations.^3^^,^^6^^,^^7^^,^^8^^,^^9^ Among these, storage is likely the most critical, as it directly reflects water availability and supports effective water regulation. To enhance global water monitoring, NASA has launched the Global Water Reservoir product, which leverages remote sensing data from the Moderate Resolution Imaging Spectroradiometer (MODIS) and the Visible Infrared Imaging Radiometer Suite (VIIRS).^8^ This satellite product enables water storage monitoring at 8-day and monthly intervals for 164 large reservoirs worldwide. More recently, Li et al.^9^ developed a fully validated, long-term Global Reservoir Storage (GRS) dataset, expanding coverage to 7,245 reservoirs (Figure 1A) by integrating Landsat-derived surface area estimates with remotely sensed and simulated bathymetry data. Although global surface water storage datasets have been developed,^3^^,^^6^^,^^8^^,^^9^^,^^10^ most provide relative storage changes rather than absolute storage values. GRS currently represents the most comprehensive dataset for estimating actual surface water storage. Despite these advancements, significant knowledge gaps persist—particularly regarding disparities in storage change between developed and developing countries.

Here, based on the high-quality GRS dataset, we observed a near-continuous increase in total reservoir storage from 1999 to 2018, with growth rates of 7.19 ± 0.02 km^3^/yr in developed countries and 16.91 ± 0.06 km^3^/yr in developing countries (Figures 1B and 1C). This increase was primarily driven by the construction of new reservoirs. While total storage provides insights into overall water availability, normalized storage (NS)—the ratio of actual storage to storage capacity—serves as a more effective metric for assessing the utility of reservoirs. NS accounts for differences in reservoir capacity, allowing for meaningful comparisons across regions. However, unlike total storage, NS trends revealed stark contrasts: developed countries exhibited a significant increase (0.0625 ± 0.0005 %/yr, *p* < 0.001), whereas developing countries showed a pronounced decline (−0.1255 ± 0.0010 %/yr, *p* < 0.001). This divergence suggests that, despite rising total storage, developing countries are likely facing escalating water stress.

At the national scale, developing countries exhibited lower NS values than developed countries (Figure 1D), with mean NS values of 57.42% and 65.02%, respectively. Additionally, NS values in developing countries fluctuated more significantly, as reflected by a mean coefficient of variation (CV) of 17.48%, compared to 7.88% in developed countries (Figure 1E). Developing countries were also more vulnerable to NS declines (Figure 1F). Globally, among the 139 countries with reservoirs, 105 exhibited significant NS trends. The majority of developed countries (27 out of 38) showed significant increases in NS, while over half of the developing countries (37 out of 67) experienced significant declines. These findings suggest that reservoirs in developing countries were not filling to the same level of capacity as those in developed countries, highlighting a widening gap in water storage resilience between the two groups.

Russia (919.67 km^3^), Canada (842.02 km^3^), China (680.57 km^3^), the United States (600.24 km^3^), and Brazil (546.38 km^3^) rank as the top five countries in terms of total reservoir capacity. Among them, the United States and Russia exhibited relatively stable storage variations, while Canada showed a significant NS increase at a rate of 0.1840 ± 0.0005 %/yr (*p* < 0.001). These three developed countries consistently maintained high NS levels, with long-term mean values of 70.60% (United States), 79.35% (Russia), and 77.69% (Canada), and exhibited minimal inter-annual fluctuations, as indicated by their low CV values of 4.67%, 2.63%, and 3.20%, respectively. In contrast, regarding the two leading developing countries, China exhibited a significant NS increase of 0.2185 ± 0.0015 %/yr (*p* < 0.001), whereas Brazil experienced a notable decline of 0.3980 ± 0.0040 %/yr (*p* < 0.001), indicating substantial storage loss. Despite China’s increasing trend, both countries had relatively low long-term mean NS values—54.28% for China and 65.45% for Brazil—accompanied by large inter-annual variations, as reflected by their CV values of 10.11% and 11.81%, respectively. These fluctuations suggest that reservoirs in China and Brazil are more vulnerable to water stress compared to those in developed nations.

Approximately 80% of the global population resides in developing countries, yet these nations collectively hold less than 50% of the world’s reservoir storage capacity. Moreover, global population growth is outpacing the expansion of reservoir storage, leading to a significant decline in per-capita water storage in both developed and developing countries. Specifically, per-capita storage is decreasing at rates of −3.14 ± 1.24 m^3^/yr in developed countries (*p* < 0.05) and −1.98 ± 0.41 m^3^/yr in developing countries (*p* < 0.001). Although developed countries have a much higher average per-capita storage (1,540 m^3^) compared to developing countries (378 m^3^), the relative loss rates are 0.20 ± 0.08 and 0.52 ± 0.11 %/yr, respectively. This more rapid decline in developing countries underscores their growing water scarcity challenges. The shrinking of surface water resources not only increases vulnerability to water stress but also poses significant obstacles to social and economic development, particularly in regions already struggling with water insecurity.

Our findings indicate that reservoirs in developing countries have become increasingly less full in the 21st century, despite a continued expansion in total storage capacity. Globally, per-capita reservoir water storage is highly uneven, with developing countries possessing only 24.55% of the per-capita storage available in developed countries, underscoring the disproportionate access to water resources. Additionally, the relative decline in per-capita storage in developing countries is 2.6 times greater than that observed in developed nations, further exacerbating water scarcity in regions already facing high demand and limited supply. These disparities highlight the pressing challenges of managing finite water resources through reservoir infrastructure, particularly in developing countries that are more vulnerable to climate variability, rapid population growth, and economic constraints. Bridging the widening water security gap requires proactive strategies, including enhanced reservoir operations, strengthened water conservation, and the adoption of alternative water management approaches such as groundwater recharge, rainwater harvesting, and equitable policy frameworks.

Addressing these challenges aligns closely with the objectives of United Nations Sustainable Development Goal 6 (SDG 6), which seeks to ensure the availability and sustainable management of water and sanitation for all. Specifically, the observed trends highlight limited progress toward target 6.4, which emphasizes improving water-use efficiency and ensuring sustainable withdrawals to reduce water stress. Integrating satellite-based monitoring tools, such as the GRS dataset, into national and international water governance systems can support data-driven decision-making and accelerate progress toward achieving SDG 6.

## Funding and acknowledgments

This work was supported by the 10.13039/501100001809National Natural Science Foundation of China (42201349), the 10.13039/501100012166National Key Research and Development Program of China (2024YFF1307700), the 10.13039/501100002865Chongqing Municipal Science and Technology Bureau (cstc2024ycjh-bgzxm0043), and the 10.13039/501100002367Chinese Academy of Sciences Pioneer Initiative Talents Program. The GRS dataset is publicly available at https://doi.org/10.5281/zenodo.7855477.

## Declaration of interests

The authors declare no competing interests.
